# Some Idle Thoughts and Lightweight Observations

**Published:** 1911-09

**Authors:** 


### Some Idle Thoughts and Lightweight Observations.

We have recently been considerably under the weather, and have had an opportunity of doing some thinking—a very good thing for the average doctor to do, provided he thinks along pleasant lines when he is sick, and a very bad thing for a sick man to do when he allows his mind to dominate him and fix up all sorts of pictures for his mental vision that are calculated to depress him and retard his recovery.

While it is not very natural for a sick man to be boiling over with joy and mirthfulness, there are certain conditions of physical derangement that are so closely connected with mental despondency and foreboding (and probably the cause of the same) that when a patient gets well "down in the mouth" we naturally expect to find the liver torpid, the digestion impaired, and general auto-infection from an atonic intestinal tract. There are other conditions that lead up to this unhappy frame of mind, and, among them, too much steady work, and too little relaxation or the right kind of rest.

Now, during our six or eight weeks of trying to get the evil spirits out of our liver, trying to tone up our colonic machinery, making an effort to eat without appetite, courting sleep that would not come, and making a fool of ourself trying to be cheerful under such conditions, we awoke to the fact that we were more *tired* than sick, and that liver, stomach and the whole intestinal race-track were on strike.

It is true that we did our full duty with calomel, podophyllin, and the salines, all of which performed in the orthodox manner; but we somehow got thinner, weaker and mentally bluer. For the past four weeks there has been something doing down here in the way of solar stunts, all of which we caught, in addition to our present holdings. So about three weeks ago we had about reached the limit, and felt that we were in a losing game, when a very dear medical friend told us seriously to leave Richmond *that day.* Go anywhere to get in the country, regardless of accommodations—change the conditions, get out of the city heat and noise, get down to the simple life of the cornbread and buttermilk-fed rustic.

We didn't feel able or cheerful enough to struggle with the conditions he pictured, but our wife, a woman of great strength of character, and a great stickler for carrying out *other doctors'* directions, when they apply to *ourself,* simply said, "You go today, and I go with you to keep you there." Our heart sank within our perspiring body, but we knew there was no way out of it; so we compromised by her agreeing to go to our own little ranch a few miles from town in the woods. This was very fortunate, for the trip was easy, and we were not dependent upon the whims of strangers for permission to do as we pleased. We just loaded our old one-lunger Cadillac with supplies and "restoratives," and C. A. B., Jr., ran us out in good time. We had a camper's cottage, with a fair equipment of beds, furniture and a lot of staple supplies, but the best thing out there was our lone old friend, John Covington, an old Virginia negro, the "old school" type of the polite, cheerful darkey of by-gone days. John occupied his little cottage, near our own, as caretaker and general sentinel.

We found him there, feeding the chickens and dodging about preparatory to "gittin' his supper." The sight of this cheerful old fellow, so trustful in Providence to provide for him, without care, and absolutely contented with his humble surroundings, gave us a feeling of rest and tranquility hard to realize.

As we looked at him, we soliloquized somewhat thusly: "This old negro is getting more out of life than we are. He is without any worry over tomorrow; he can eat a full meal of plain food, which satisfies him, and can sleep like a log all night long. Now, who is the philosopher, and who the fool, when John Covington and Dr. Bryce toss up to settle the question?"

Well, we will leave this an open question, and say that there is a middle ground somewhere between the extremes we have drawn, that more of us might occupy to advantage. We have found our sequestered little place helpful, remedial and restful, and are out here now, which accounts for this imposition upon our readers. We have nothing else to do, and must fill *The Clinic* up with something. Besides, it may be that others, like ourselves, will get to thinking, and will find that it doesn't pay to worry and bother over matters that are of no moment in the long run. There is also something of a medical side to this little rumination of ours. We have been thinking that medication may be overdone, that we can help nature a good deal in her own way, and that, in many instances, a perfect surrender to the natural life and primitive conditions is the only chance for restoration of the patient who has overdrawn his nerve account.

The medical man, of all others, is at a disadvantage when he gets a nervous jag on himself from work or worry; for he begins to analyze his symptoms from the wrong viewpoint usually, and burdens himself with "what's coming." In other words, he knows too much about symptoms, and not a thing about his own condition.

The next trouble is his inability to escape from the insistent ones who must see him for just a minute, or just one word over the 'phone, as long as he tries to live in his own home. A medical man can not get rest and tranquility at his home if he has reached that state of nerve tension that makes him want to kick the dog, "cuss" the mail carrier, or puts cotton in his ears when his little daughter regales him with that delightful melody on the discordant piano, "Everybody Works but Father."

The time has come for him to drop his M. D.; be sure to affix "Mister" to his name, and absolutely hide his medical identity; then provide himself with a lot of blood-and-thunder dime novels, nothing stronger, and engage board with Farmer Corntassel, whose land extends to the "crick" that is full of perch, with good "fishin' holes every whar."

Now, if you are suffering from anorexia and insomnia, as you are most likely to be, you have the best opportunity in the world for ascertaining the remedy by studying the habits and antics of old Hayseed, Esq. This picturesque type of rustic humanity, if carefully studied and half-way analyzed, will certainly make a neurasthenic forget himself. Some years ago we roosted for several weeks with one, and grew younger while getting strength under his hospitable roof. This old fellow was past sixty, but as tough and wiry as a mustang. He was up in the mornings ahead of us, however soon we might arise, and, with his run-down shoes, blue "britches," all frazzled at the bottoms, and held up with his galluses crossed, and one of them pinned on to his "britches" with a ten-penny nail for lack of a button, his shirt unbuttoned at the neck, his old ropy beard swinging around like a billy-goat's, and his head crowned with a big turned-up kind of a bark hat; he reminded us of the pictures of Uncle Sam—impudent, independent, good-hearted, grand old cuss—the very man to make you forget all of your imaginary ailments and catch the infection of rising spirits. We determined to see about what his exercises consisted of before breakfast, and, getting up before sunrise one morning, we learned that he was down at the "cupen" (they meant cow-pen, or where they milked the cows); so we went down to this combination of barn and shelters, but, before we got there we heard a great rumbling, kicking, scuffling and "cussing," and just as we got there we saw the old fellow, bare-headed, galloping around the corner of one of the sheds, with a pretty big calf by the ear with his left hand and twisting his tail with the other, to make him trot along. The old fellow was white and red in the face alternately, "cussing" the calf whenever he could get breath, and the calf was bellowing and yanking him along at a pretty lively gait. He finally slipped the calf into another pen, slammed the gate together and securely fastened it. The old fellow looked rather sheepish when he saw that we had witnessed his performance, and asked us if we had enjoyed the fun very much. We told him it was the best we had ever witnessed. "Well, by gosh," said he, "ef you tuk a little more exercise like that yerself, you wouldn't be huntin' around so durned much for an appetite." He was hot in the collar all right, but when breakfast was ready he was as genial as a mint julep, and helped us twice to fried chicken.

These people have no time to think about imaginary ills; they have to hustle to live, and it is a blessing. The same day old man D. had the tussle with the calf, the hogs got into his watermelon patch and the bees swarmed in the afternoon. You can not have melancholy, want of appetite, or sleeplessness under the "strenuosity" of such a life.

Now, doctor, instead of investing in some gold mine or get-rich-quick scheme, put a few hundred dollars in a few suburban acres, where you can hide and rest when the evil days come, and when the natural growth of the city comes to you, as it surely will and always has done, you will have laid up a treasure that will pay you in health, happiness and many times the amount of dollars expended.—Bryce, in *Southern Clinic.*

